# Stability and Change of Adolescents’ Aggressive Behavior in Residential Youth Care

**DOI:** 10.1007/s10566-017-9425-y

**Published:** 2017-11-13

**Authors:** E. M. A. Eltink, J. Ten Hoeve, T. De Jongh, G. H. P. Van der Helm, I. B. Wissink, G. J. J. M. Stams

**Affiliations:** 1 Department of Forensic Child and Youth Care Sciences, University of Amsterdam, P.O. Box 15780, 1001 NG Amsterdam, The Netherlands; 2 Department of Institute of Social Work, University of Applied Sciences of Utrecht, Utrecht, The Netherlands; 3 Youth Expert Centre, Leiden University of Professional Sciences, Leiden, The Netherlands

**Keywords:** Aggression, Repression, Residential youth care, Aggressive behavior, Deprivation hypothesis, Import hypothesis

## Abstract

**Background:**

Aggression in residential youth care institutions is a frequent problem.

**Objective:**

The present short-term longitudinal study examined individual and institutional predictors of aggression in a group of 198 adolescents placed in open, semi-secure and secure residential institutions from the perspective of the importation and deprivation model.

**Methods:**

A total of 198 adolescents in residential youth care filled in questionnaires regarding group climate and aggression with a 3 month interval. Hierarchical multiple regression analyses were performed to test the degree to which individual and contextual factors predict aggression.

**Results:**

Very limited support was found for the effect of contextual factors; only repression showed a trend, predicting direct aggression, while gender composition of the living groups yielded a small effect. Girls placed in same-gender groups showed lower levels of indirect (relational) aggression compared to adolescents placed in mixed-gender or boys-only groups, even when controlled for gender and initial levels of aggression. Type of institution (i.e., level of security) did not predict differences in aggression. In particular individual characteristics of the adolescents were associated with later aggression, including initial levels of aggression, showing substantial 3 months stability, age and gender of the adolescents.

**Conclusions:**

These findings are in line with research showing that aggression is relatively stable. Very limited support for environmental effects was found.

## Introduction

Residential youth care includes a variety of institutions in which adolescents live in groups of 6–12 adolescents and receive treatment (Bastiaanssen et al. 2012; Barth 2002; Frensch and Cameron 2002). In the Netherlands, residential youth care is considered to be a last resort, and it is the most expensive and restrictive type of care (Harder et al. 2012). Residential youth care institutions can be distinguished by their level of restrictiveness. In open institutions adolescents attend their school and leisure activities outside the institution. Semi-secure residential institutions offer care, schooling and treatment for young people with the most serious emotional and behavioral problems (Harder 2011), which may be enforced by civil law. In these institutions adolescents ‘work’ gradually towards more privileges, including leave to visit family or to attend school outside of the institution. In secure (correctional) institutions adolescent live within the confinement of the institution, and leave is an exception rather than the rule.

In the Netherlands, adolescents in open, semi-secure and secure institutions live in small groups (Bastiaanssen et al. 2012), where group climate is thought to have a direct effect on adolescents’ development and is also assumed to moderate treatment effects (Marshall and Burton 2010; Schubert et al. 2012; Van der Helm 2011). Group climate in residential youth care has recently been defined as ‘the quality of the social and physical environment in terms of the provision of sufficient and necessary conditions for physical and mental health, well-being, contact and personal growth of the residents, with respect for their dignity and human rights as well as (if not restricted by judicial measures) their personal autonomy, aimed at recovery and  successful participation in society’ (Stams and Van der Helm, in press). Several dimensions of group climate emerge in scientific literature, which from a rehabilitative perspective may be considered as positive (e.g., support from staff, opportunities for growth, and structure), negative (e.g., repression by staff), or which may turn out to have both a positive side (e.g., peer support) and negative side (e.g., deviant peer affiliation) depending on the composition of the group of adolescents/inmates (i.e., group atmosphere) (Boone et al. 2016; de Valk et al. 2016; Tonkin 2015; Van der Helm et al. 2011).

Aggression in residential youth care institutions is a frequent problem (Barzman et al. 2011; Cornaggia et al. 2011). It may lead to hospitalization, or can be provoked by the conditions of hospitalization itself (Papadopoulos et al. 2011), which can prolong inpatient stay (Baeza et al. 2013). The persistent aggressive behavior problems of juveniles in residential youth care seem to lead to higher levels of behavioral control by staff, which may result in repression and a coercive cycle of interaction (de Valk et al. 2016; Vermaes and Nijhof 2014).

Aggression is most often defined as behavior that is intended to injure or harm someone physically or psychologically (Baron and Richardson 2004; Bushman and Anderson 2001). It is considered to be the result of a complex interaction between personal, interpersonal, and circumstantial variables (Allen et al. 2017; Mendes et al. 2009). The defining characteristic of aggression is the intent to cause harm to another person, but the form that aggression takes can be either direct, such as a physical confrontation with the victim, or indirect, often referred to as relational aggression (Warren et al. 2011).

In residential youth care, aggression may be expressed verbally, such as bullying, or physically (Barter et al. 2004; Sekol 2013; de Decker et al. 2017). Notably, from research on bullying in prisons, it can be derived that exposure to frequent aggression and perceived threat of aggression may contribute to both a normalization of and desensitization to aggression. Additionally, the interaction between individual characteristics of inmates (i.e., aggressive tendencies) and the adverse (i.e., aggression-eliciting) prison environment reinforces aggression (Ireland 2002; Turner and Ireland 2010). Therefore, the development of aggression in residential institutions has been explained from the perspective of the importation and deprivation model (DeLisi et al. 2011; Gover et al. 2000; Jiang and Fisher-Giorlando 2002).

The importation model explains aggressive behavior from characteristics, experiences, and attitudes of the residents themselves, in particular tendencies to behave aggressively (Kuanling et al. 2008; Gover et al. 2000), while the deprivation model explains aggressive behavior of the residents (e.g., prisoners, justice-involved youth, adolescents staying in residential care because of serious psychopathology or lack of a safe home) from deprivation, repression and the loss of autonomy associated with detention (Sykes 1958) or staying in an institution (Souverein et al. 2013).

### The Importation Model: A Focus on Adolescents’ Characteristics

Adolescents in residential youth care generally have limited ability to react adequately in social problematic situations due to problems in social information processing, which may result in aggression as a problem solving strategy (Arsenio et al. 2009; Eltink et al. 2015; Nas et al. 2005; Van der Helm et al. 2013).Youth in residential care constitutes a high-risk group for psychological, psychiatric, educational, social, health, and behavioral problems (e.g., Attar-Schwartz 2008, 2009; Colins et al. 2013). These adolescents often have a history of neglect and/or abuse (Van Dam et al. 2010), while being a victim of abuse is a risk factor for becoming a perpetrator of abuse later in life (the ‘cycle of violence’; Widom 1989), or future adolescent offending (Asscher et al. 2015). Notably, Dodge (2006) showed that past experiences of insecurity, such as long-term exposure to violent environments or frequent placements in institutions can lead to heightened vigilance, hostile and distrustful thoughts, which can evoke aggression (Sato et al. 2009).

Gender differences in aggression are well established, with boys exhibiting substantially more direct aggression and girls showing slightly more indirect aggression (Bjorkqvist 2017; Card et al. 2008). Also in residential youth care, boys have been shown to exhibit more externalizing problems, including aggressive and delinquent behaviors, than girls (e.g., Attar-Schwartz 2008; Glisson et al. 2002; Schiff and Benbenishty 2006). Aggressive incidents in adolescent inpatient facilities have particularly been associated with male sex (Barton et al. 2001). Piquero et al. (2012) reviewed the literature on the development of aggression, and concluded that the continuity of childhood, adolescent, and adult problem behavior is “one of the few ‘knowns’ in criminology”. Piquero et al. (2012) concluded that aggression is rather stable in childhood and throughout adolescence, in particular in the most aggressive adolescents and persistent juvenile offenders, but begins to gradually decrease for most persons in early adulthood, which concurs with the results of general populations studies showing that problem behavior, including aggression, declines with age (Bongers et al. 2003). Although aggressive behavior should be distinguished from delinquent behavior both in etiology and prevalence rates (Bongers et al. 2003, 2004; Dishion and Patterson 2006), the age-crime curve (Hirschi and Gottfredson 1983) suggests a curvilinear relation for aggressive forms of delinquent behavior, with a peak in late adolescence and a decline afterwards (Fagan and Western 2005).

Baker et al. (2005) found that younger children in residential treatment reported more aggression than older children. This result is consistent with studies of Cunningham and Sorensen (2006, 2007), DeLisi et al. (2004), and Vassallo et al. (2016), who showed that younger age was predictive of aggressive behavior within residential institutions. Also, Eltink et al. (2015) found that younger adolescents in residential youth care reported more problematic reactions to aggression eliciting situations than did older adolescents. Van den Tillaart et al. (2017) found that age was not a predictor of aggressive behavior of adolescents in residential care. However, Manso et al. (2011) showed that younger children found it easier to adjust to residential care than older children, resulting in more hostility in the latter group. We conclude that most studies in residential youth care show a negative relation between age and aggression.

Finally research showed that there are individual differences between adolescents in secure, semi-secure and open institutions. Vermaes and Nijhof (2014) found that adolescents in semi-secure residential youth care had lower self-esteem, impaired emotion regulation and showed more antisocial and aggressive externalizing problems, whereas adolescents in open youth care showed more internalizing problems. In secure institutions, as compared to semi-secure institutions, aggressive behavior, autism, substance abuse and personality disorders are more common (Smeets 2014).

### The Deprivation Model: Negative Aspects of Residential Youth Institutions

Negative influences of residential institutions are thought to result from the effects of staying in a residential institution itself (Dye 2010; White et al. 2010). Sykes (1958) describes deprivation in prisons as the loss of freedom, goods, services, autonomy, security, and frequent contact with family and friends due to the nature of the institution as such. Just as life in prison, residential living of any kind means that the whole personality of a young person is involved in a more or less inescapable social system (Elliot and Thompson 1991), even so when adolescent males and females are placed in same-gender or mixed-gender groups. In general, boys and girls live in mixed-gender groups, which are thought to have developmental value, for instance, from the perspective of sexual development (Connolly et al. 2004). However, girls and boys are often placed in same-gender groups when entering a residential institution, which may be for good reasons if a same-gender group provides more protection and better outcomes, but which still should be considered as a restrictive measure, in particular because there is still not much direct empirical evidence for the positive effects of same-gender placement. Notably, the negative effects of antisocial (aggressive) peer association through deviancy training may even be greater in the more homogenous same-gender groups, in particular boys, than the more heterogeneous mixed-gender groups (Dishion et al. 1999; Dishion et al. 2001).

In line with the deprivation model (Goffman 1961; Sykes 1958), adolescents in residential youth care often act out aggressively in response to frustrating conditions within their residential facilities, which may take the form of bullying (Sekol 2013). Harer and Steffensmeier (1996) found the security level of the institution to have a positive relation with aggressive behavior, although it should be kept in mind that the more aggressive youths tend to be placed in (semi)secure institutions (Vermaes and Nijhof 2014). Gover et al. (2000) and Harer and Steffensmeier (1996) found that higher security levels can lead to poor adjustment and aggressive behavior. On the contrary, Camp and Gaes (2005) found no relation between aggressive behavior and type of institution (with varying security levels), whereas Davidson-Arad (2005) found that aggressive behavior was more common in open institutions than in secure juvenile offender institutions. Aggressive behavior during child and adolescent hospitalization has also been related to an increasing length of stay (Dean et al. 2008). Recently, longer stay in residential care was also found to be related to more aggression incidents (Van den Tillaart et al. 2017).

Coercion is thought to be part of the structure and control necessary in a residential context to prevent chaos (Souverein et al. 2013), but it can turn into repression due to extreme power imbalance (Souverein et al. 2013; Zimbardo 2007). Repression in residential youth care has recently been defined as authorities intentionally acting in a way that harms the youth, or authorities unlawfully or arbitrarily depriving the youth of liberty or autonomy (de Valk et al. 2016), which has been shown to result in antisocial behavior of the residents (Heynen et al. 2016; Pritikin 2009). Staff often responds to such antisocial behavior by intensifying repression (Davidson-Arad and Golan 2007; Van der Helm et al. 2011), which contributes to a loss of the sense of control and autonomy of the residents, provoking new aggressive behavior (Van der Helm 2011).

### Current Study

The current short-term longitudinal study examined predictors of self-reported aggression in residential youth care from the perspective of the importation model (individual characteristics of the adolescents that are thought to be related to aggression) and deprivation model (institutional characteristics that are thought to be related to aggression of the adolescents). We examined the association between characteristics of the adolescents and their level of aggression after 3 months. We hypothesized that age of adolescents would be negatively associated with later aggression, that boys would report more direct aggression and girls more indirect aggression at T2, and that aggression at T1 would be positively associated with aggression at T2. We focused on the impact of the social environment by examining differences in aggression related to placement in a same-gender group versus mixed-gender group, the degree of restrictiveness of the institution (open/semi-secure and secure), length of stay and repression. We expected a longer stay in the residential institution to be associated with more aggression over time, adolescents in semi-secure and secure settings to report more aggression than adolescents in open settings at T2, and repression to be positively associated with aggression at T2. Environmental influences were tested in multiple hierarchical regression analyses by first controlling for individual (import) characteristics of the juveniles. Finally, we examined whether perceived repression would be differentially related to aggression in the three types of institutions and same-sex and mixed-gender groups, because both levels of repression and aggression may differ between semi-secure, secure and open institutions as well as in same-sex and mixed-gender groups.

## Method

### Participants

A total of 198 juveniles participated in our study, with complete data at T1 and T2, 3 months after T1. It is a sample of convenience of all juveniles who were available at the time to participate in the study. Of the sample, 49 juveniles were placed in open youth institutions, 106 in semi-secure youth institutions, and 43 juveniles were placed in youth prisons (secure youth institutions). From the population (*N* = 198) 130 participants were boys and 68 were girls. The average age of the boys (*n* = 130) was 16.2 years (*SD* = 1.83) and the average age of the girls (*n* = 68) was 15.8 years (*SD* = 1.17).

### Procedure

Participants were asked to completed the questionnaires. All respondents participated voluntarily, signed an informed consent declaration, and were assured that their answers would be treated confidentially and processed anonymously, being accessed only by the researchers. All names on the questionnaires were deleted and given a code number in SPSS. To protect the privacy of the respondents, researchers had no access to the names. All interviews and questionnaires were administered by specially trained graduate students of the Leiden University of Applied Sciences (bachelor of social work and master youth care) and the University of Amsterdam (Department of Forensic Child and Youth Care Sciences). The study protocol was approved by the ethical committee of the Leiden University of Applied Sciences. None of the authors have a conflict of interest.

### Measures

The perception of repressive group climate was measured by means of the repression scale of the Prison Group Climate Instrument (PGCI; Van der Helm et al. 2011). Repression (7 items) is rated on a 5-point Likert-type scale, ranging from 1 = *I do not agree* to 5 = *I totally agree,* and assesses perceptions of strictness and control, unfair and haphazard rules, and lack of flexibility at the living group. An example of a repression item is “You have to ask permission for everything here.” In the validation study of the PGCI, the factor *repression* proved to be reliable, with internal consistency reliability of α = .76 (Van der Helm 2011). The present study shows a reliability of α = .80.

Aggression was measured with the Buss Durkee Hostility Inventory-Dutch version (BDHI-D; Lange et al. 2005). The BDHI-D consists of 40 items (true/false) and 3 scales: direct aggression, indirect aggression and social desirability. The scale for social desirability has not been consistent nor reliable, and was therefore not used in the present study. Direct aggression items consist of a combination of physical and verbal aggression; anger and hostility are concepts of indirect aggression. An example of a direct aggression item is “If somebody hits me first, I let him have it.” An example of an indirect aggression item is “I’m often angry, without other people knowing”. The internal consistency reliability in the validation study of Lange et al. (1995) on the scale for direct aggression was α = .77, and for indirect aggression α = .79. The present study showed a reliability for direct aggression of α = .80 at T1 and α = .77 at T2. For indirect aggression the present study showed a reliability of α = .80 at T1 and α = .82 at T2.

### Analyses

We first conducted preliminary univariate analyses, after imputation of missing data through Expectation Maximization (EM), computing simple correlations between all variables of interest, that is, (static) individual adolescent characteristics, length of stay, gender group, type of institution, repression and aggression at Time 1 (T1) and Time 2 (T2). Subsequently, we computed intraclass correlations (ICC) by means of a multilevel random intercept-only model (null-model) to examine whether there was significance between living group variance in both indirect and direct aggression. Because the intraclass correlations were zero, and therefore non-significant, we decided to conduct standard hierarchical multiple regression analyses. The effects of variables that are included on later steps in the model are only meaningful if controlled for the variables entered in previous steps given the theoretical priority of repression above level of security, which is only a static factor. Moreover, testing environmental influences requires that individual characteristics of the adolescents are controlled for in order to rule out the alternative explanation that the effects of contextual factors on aggression may be explained by individual factors, that is, characteristics of the adolescents instead of institutional characteristics.

We entered variables in the following eight consecutive steps, starting with adolescents’ individual characteristics (first static, subsequently dynamic), followed by contextual variables: step 1, static adolescent characteristics (age and gender); step 2, indirect and direct aggression at T1; step 3, length of stay; step 4, gender group (the dummy variables boys-only and girls-only); step 5, type of institution (the dummy variables secure and open institution), step 6, repression at T1; step 7, the interaction between repression and gender group; step 8, the interaction between repression and type of institution.

## Results

### Preliminary Results

It can be derived from Table 1 that most variables were intercorrelated (mostly weak correlations). Girls were underrepresented in secure institutions (*r* = − .25), and showed less indirect aggression at T1 (*r* = − .19) and T2 (*r* = − .15). In boys-only groups and secure institutions adolescents were older (*r* = .19 and *r* = .51, respectively), whereas in semi-secure institutions adolescents were somewhat younger (*r* = − .36). Furthermore, age was positively associated with direct and indirect aggression at T1 (*r* = .15 and *r* = .18, respectively), and direct and indirect aggression at T2 (*r* = .15 and *r* = .17, respectively). Adolescents in boys-only groups had a shorter stay in secure institutions (*r* = − .21 and *r* = − .48) and length of stay was longer in semi-secure institutions (*r* = .27). In addition, length of stay was negatively associated with both indirect aggression at T1 and T2 (*r* = − .16). Girls-only groups were overrepresented in semi-secure institutions (*r* = .22) and underrepresented in secure institutions (*r* = − .14). Furthermore, lower levels of indirect aggression were reported at T1 and T2 in girls-only groups (*r* = − .20 and *r* = − .22, respectively). Boys-only groups were overrepresented in secure institutions (*r* = .41), and reported higher levels of indirect aggression at T1 and T2 (*r* = .21 and *r* = .16). Adolescents in semi-secure institutions reported less indirect aggression at T1 and T2 (*r* = − .18 and − .22, respectively) as well as less direct aggression at T2 (*r* = − .15), and more repression at T1 (*r* = .35) than adolescents in the other institutions. Adolescents in secure institutions reported more indirect aggression at T1 and T2 (*r* = .21 and *r* = .17, respectively) than adolescents in the other institutions. Direct aggression at T1 was positively correlated with indirect aggression at T1 (*r* = .43), and direct and indirect aggression at T2 (*r* = 72, and *r* = .35, respectively). Furthermore, direct aggression at T1 was negatively associated with repression at T1 (*r* = − .24). Indirect aggression at T1 was positively correlated with direct and indirect aggression at T2 (*r* = .34 and *r* = .73, respectively), and negatively correlated with repression at T1 (*r* = − .31). Finally, repression at T1 was negatively correlated with indirect aggression at T2 (*r* = − .34), while direct and indirect aggression were positively correlated at T2 (*r* = .42).

**Table 1:** Two-tailed Pearson’s correlations of all variables

	*M*	*SD*	1	2	3	4	5	6	7	8	9	10	11	12
1. Gender (1 = girl)	.34	.48	1											
2. Age	16.05	1.64	− .11	1										
3. Length of stay (1 = long)	.93	.24	.14	− .07	1									
4. Girls group	.23	.42	.72***	− .04	.09	1								
5. Boys group	.49	.50	− .72***	.19**	− .21**	− .54***	1							
6. Semi secure	.53	.50	.08	− .36***	.27***	.22**	− .09	1						
7. Secure	.22	.41	− .25***	.51***	− .48**	− .14*	.41***	− .57***	1					
8. Direct aggression T1	1.76	.41	− .07	.15*	.02	− .02	.06	− .08	.00	1				
9. Indirect aggression T1	2.09	.42	− .19**	.18*	− .16*	− .20**	.21**	− .18*	.21**	.43***	1			
10. Repression	3.36	.86	− .08	− .05	.10	.08	.12	.35***	.01	− .24**	− .31***	1		
11. Direct aggression T2	.174	.39	− .06	.15*	− .09	− .01	.07	− .15*	.08	.72***	.34***	− .13	1	
12. Indirect aggression T2	2.08	.42	− .15*	.17*	− .16*	− .22**	.16*	− .22**	.17*	.35***	.73***	− .34***	.42***	1

*N* = 198

* *p* < .05; ** *p* < .01; *** *p* < .001

### Multiple Hierarchical Regression Analysis

Two hierarchical multiple regressions were used to predict indirect and direct aggression from adolescents’ and contextual characteristics, respectively. The entry order of the variables permits an examination as to whether the variables of interest account for any additional variance in the criterion variable that is not explained by previously entered predictors.

Table 2 shows the results of the hierarchical multiple regression analysis for direct aggression at T2. The regression equation was significant, *F*(14, 183) = 15.55, *p* < .001, the predictors explained 54% of the variance in direct aggression at T2. Age of the adolescent (*β* = .15) and direct aggression at T1 (*β* = .70) were both positively associated with direct aggression at T2, whereas length of stay (*β* = − .10) was negatively associated with direct aggression at T2. Finally, repression (*β* = .10) just failed to reach significance, showing a trend indicating that higher levels of repression at T1 predicted more direct aggression at T2.

**Table 2:** Multiple hierarchical regression analysis: direct aggression

Predictors	*R*	*R* ^2^	*ΔR* ^2^	*ΔF*	*β*	*t*
Static individual characteristics	.16	.03	.03	2.46^+^		
Sex of the adolescent					− .04	− 0.57
Age of the adolescent					.15	2.07*
Aggression at time 1	.72	.52	.49	99.38***		
Direct aggression					.70	12.63***
Indirect aggression					.03	0.55
Short versus long stay	.73	.53	.01	3.92*	− .10	− 1.98*
Gender group	.73	.53	.00	0.08		
Girls-only					.01	0.18
Boys-only					.03	0.35
Type of institution	.73	.53	.00	0.85		
Secure					− .03	− 0.41
Semi-secure					− .09	− 1.28
Repression at time 1	.74	.54	.01	2.96^+^	.10	1.72^+^
Interaction: repression × gender group	.74	.54	.00	0.38		
Boys-only groups × repression					− .02	− 0.22
Girls-only groups × repression					− .06	− 0.84
Interaction: repression × type of institution	.74	.54	.00	0.11		
Secure × repression					.03	0.40
Semi-secure × repression					.04	0.40

*N* = 198. *F*(14, 183) = 15.55, *p* < .001

^+^ *p* < .10; * *p* < .05; ** *p* < .01; *** *p* < .001

Table 3 shows the results of the multiple hierarchical regression analysis for indirect aggression at T2. The regression equation was significant, *F*(14, 183) = 15.55, *p* < .001): the predictors explained 56% of the variance in indirect aggression at T2. Age of the adolescent (β = .16) and indirect aggression at T1 (β = .71) were both positively associated with indirect aggression at T2, whereas gender composition of the groups (β = − .16) was negatively associated with indirect aggression at T2, indicating that girls-only groups showed less indirect aggression. Finally, sex of the adolescent (β = − .13) showed a trend indicating that girls experienced less indirect aggression at T2.

**Table 3:** Multiple hierarchical regression analysis: indirect aggression

Predictors	*R*	*R* ^*2*^	*ΔR* ^*2*^	*ΔF*	*β*	*t*
Static individual characteristics	.22	.05	.05	4.74*		
Sex of the adolescent					− .13	− 1.83^+^
Age of the adolescent					.16	2.26*
Aggression at time 1	.74	.54	.49	103.94***		
Direct aggression					.04	0.77
Indirect aggression					.71	12.82***
Short versus long stay	.74	.54	.00	0.78	− .04	− 0.89
Gender group	.75	.56	.02	2.59^+^		
Girls-only					− .16	− 2.27*
Boys-only					− .01	− 0.19
Type of institution	.75	.56	.00	0.79		
Secure					− .07	− 1.21
Semi-secure					− .08	− 1.28
Repression at time 1	.75	.56	.00	2.51	− .09	− 1.58
Interaction: repression × gender group	.75	.56	.00	0.48		
Boys-only groups × repression					.05	0.68
Girls-only groups × repression					− .02	− 0.30
Interaction: repression × type of institution	.75	.56	.00	0.38		
Secure × repression					− .06	− 0.88
Semi-secure × repression					− .04	− 0.44

*N* = 198. *F*(14, 183) = 15.55, *p* < .001

^+^ *p* < .10; * *p* < .05; ** *p* < .01; *** *p* < .001

## Discussion

The present short-term longitudinal study focused on the explanation of adolescent self-reported aggression in residential youth care from the perspective of the importation and deprivation model, focusing on characteristics of the adolescents, such as initial levels of aggression, and features of the institution, including the level of security (open, semi-secure or secure institution) and repression by staff as perceived by the adolescents. Based on self-report data, we found support for the stability of aggressive behavior across a relatively short time period. Results provided very limited support for environmental influences, because multilevel analyses did not show significant context variance (i.e., between living group differences in aggression at T2), while perceived repression at T1 and length of stay (not in the expected direction) only showed a weak trend-like association with direct aggression at T2, with negligible effects. Gender composition of the living group did have a small environmental effect, which indicated that girls-only groups showed less indirect aggression at T2 compared to boys-only and mixed gender groups, even after controlling for gender (at the individual level) and initial levels of aggression. No significant effects were found for type of institution. Finally, older adolescents reported more direct and indirect aggression at T2, and there was a trend showing that girls reported less indirect aggression at T2.

Previous research on group climate and aggression in Dutch youth prisons and in a secure forensic psychiatric units for adolescents in Belgium showed that repression was unrelated to aggression (de Decker et al. 2017; Ros et al. 2013; Van der Helm 2011), which is in line with results of the present study. It has been suggested that the repression adolescents in residential youth care perceive does not differ much from the repression they may have experienced prior to their residential placement in adverse child-rearing situations, which may therefore hardly increase aggression during their stay in a highly restrictive residential environment (Anderson 2000; Bugental and Schwartz 2009; De Jong 2007; Dodge et al. 2007; Sato et al. 2009; Van Spinhoven et al. 2010).

Unlike findings in the Netherlands, Heynen et al. (2016) found perception of a more repressive group climate to be associated with higher levels of reactive (but not proactive) aggression in incarcerated juvenile offenders in Germany, which is consistent with the deprivation hypothesis. They suggested that the relation between living group climate and aggression might be affected by differences in the juvenile prison system between Germany and the Netherlands; the German juvenile prison system particularly focuses on formal education, reduction of drug use and aggression (Walter 1999), but does not target reduction of aggressive behavior by means of evidence-based offender rehabilitation programs, such as responsive aggression replacement therapy (Hoogsteder et al. 2014). Also, the repression scale of the PGCI consists of items measuring both repression and deprivation. Deprivation items loaded relatively high on the German version of the PGCI, whereas repression items loaded relatively high on the Dutch version of the PGCI (Heynen et al. 2014; Van der Helm 2011). Therefore, Heynen et al. (2016) argued that deprivation rather than repression may explain the German results.

Length of stay showed a trend-like negative association with indirect aggression, which indicated that adolescents staying longer in residential care reported slightly less indirect aggression at T2. This is in line with findings of a study by Cunningham and Sorensen (2007) in Florida (USA), showing that a longer stay in juvenile prison was associated with lower rates of aggressive behavior, but inconsistent with recent research in the Netherlands by Van den Tillaart et al. (2017), who showed that length of stay in both open and secure residential institutions was positively associated with involvement of adolescents in aggressive incidents. However, in the present study we assessed the predisposition to exhibit aggressive behavior by means of adolescent self-report instead of aggressive incidents during detention through incident registrations. It is plausible to suggest that self-perceived indirect aggression decreases with time spent in the institution, because social positioning to acquire social status (which elicits relational aggression) may decrease when relationships among adolescents at the living group progressively stabilize (Archer and Coyne 2005).

The present study found no evidence that adolescents in semi-secure and secure institutions showed higher levels of aggression after a 3-month period than adolescents placed in open institutions after controlling for characteristics of the adolescents, including initial levels of aggression. This finding is in line with results from the study by Van den Tillaart et al. (2017), who also did not find differences in aggression between open, semi-secure and secure institutions in The Netherlands. Notably Davidson-Arad (2005) compared three types of juvenile correctional facilities in Israel, and found that direct aggression (i.e., violent misconduct) was more frequent in the more open institutions compared to closed institutions. More direct aggression in the open institutions was explained by reduced supervision, while adolescents in these open institutions still were thought to have insufficient coping strategies to manage their anger and frustration. However, David-Arad’s study was cross-sectional and did not control for initial levels of direct aggression. In the present study, we also found lower levels of both indirect and direct aggression in semi-secure institutions in the univariate analyses, but these differences disappeared after taking individual characteristics of the adolescents into account. For instance, aggression at T1 (as an individual characteristic) was strongly associated with aggression at T2, which is consistent with studies showing that aggression is rather stable in high risk youth, and is often more induced by individual (including genetic) than environmental factors (Fairchild et al. 2013; Niv et al. 2013; Moffitt 1993; Tremblay 2003, 2010).

To conclude, it seems that perceived repression by adolescents in residential youth care does not, or hardly, affect their aggressive behavior. However, De Swart et al. (2012) showed in their meta-analysis that residential youth care might reduce aggressive behavior of adolescents if they receive evidence-based treatment, mostly Cognitive Behavior Therapy (CBT), targeting aggressive and delinquent behavior (see Andrews and Bonta 2010). For instance, Hoogsteder et al. (2014) showed that Responsive Aggression Regulation Therapy (Re-Art) proved to be effective in reducing aggression in adolescents placed in a Dutch youth prison and even reducing recidivism. If repression generally does not have a direct effect on aggression (as a predictor), future studies should in particular examine if, and to what extent, repression might have a negative effect on the effectiveness of treatment targeting aggression (as a moderator). To date, no such studies have been conducted.

The present study found a positive relation between age of the adolescent and aggression, both at T1 and T2, which is not consistent with our expectation, given that most studies found a negative association between age and aggression in residential youth care. It should be noted that the association between age and antisocial behavior (i.e., aggression and delinquent behaviors) tends to be rather complex, and may vary across outcome and population (Fagan and Western 2005). Juveniles in open, semi-secure and secure residential youth care institutions represent a high risk group with troubled backgrounds and high levels of psychopathology (e.g., Colins et al. 2013; Nijhof 2011; Trout et al. 2008). It is not unlikely that antisocial behavior in this highly disturbed group of adolescents peaks in late adolescence instead of early or middle adolescence, and starts to decline after late adolescence or even young adulthood (Loeber et al. 2012), which may explain the positive association between age and aggressive behavior in the present study.

Although in general girls tend to show lower levels of direct aggression than boys (Card et al. 2008; Ostrov and Godleski 2010), highly disturbed girls entering juvenile justice institutions in The Netherlands have been found to show similar levels of psychopathology, including aggression (Hamerlynck et al. 2008; Nijhof 2011). Notably, girls are less often incarcerated compared to boys, but incarcerated girls’ problems tend to be more severe than those of boys (‘Gender Paradox’; Zahn et al. 2009). In line with the study by Hamerlynck and other studies highlighting serious behavior problems in (justice-involved) girls in residential youth care (Van Vugt et al. 2014), we did not find differences in direct aggression between boys and girls in the present study. However, there was a trend indicating that girls rated somewhat lower on self-reported indirect aggression, which concurs with results from the meta-analysis of Card et al. (2008), who also found that girls did score lower on self-reported indirect aggression compared to boys, although overall (independent of assessment method) girls showed slightly more indirect aggression than did boys. We therefore should be cautious in the interpretation of our study results, in particular because in a review on the effects of complex trauma, Ford et al. (2012) found relational aggression to be a (maladaptive) coping strategy of girls in secure residential settings, in particular in those with a history of sexual abuse (Cullerton-Sen et al. 2008).

Additionally, we found less indirect aggression in girls-only groups than in mixed gender or boys-only groups, even when controlled for individual gender differences in aggression at T2, and initial levels of aggression. First, because girls have been found to display a stronger relational orientation than boys (Taylor et al. 2000; Zahn-Waxler et al. 2005), and as they tend to particularly emphasize the importance of harmonious peer relationships in residential youth care (Mathys et al. 2013), they might derive more support from their relationships with other girls in same-gender living groups, reducing levels of indirect (i.e., relational) aggression. Moreover, they might find it easier to discuss gender-specific recovery issues in same-gender groups. Second, group workers may find it less difficult to establish a positive working alliance with girls in same-gender groups than in mixed gender groups, thus creating a more supportive group environment for these girls in same-gender groups. This adds to the findings of Lanctôt et al. (2012), who showed that group workers in semi-secure care found it more difficult to work with girls than boys and building a good working alliance with them.

The present study has some limitations. First, the BDHI-D was used to assess aggression because the instrument showed no underreporting of aggression in a group of serious juvenile delinquents in the Netherlands (Breuk et al. 2007), but the BDHI-D has two drawbacks. The dichotomous ‘true’ or ‘false’ items may lead to lack of variance, and most items of the BDHI-D assess rather static (trait-like) tendencies to show aggressive behavior instead of a more dynamic (state-like) assessment of aggression, which limits possibilities to find significant changes in aggression over time. A second limitation is that the environmental variance might be somewhat circumscribed in the way it is measured (mostly dummy variables)—and perhaps also in reality—if the residential facilities do not show much differences, for instance, if they are mostly well-functioning. A third limitation is that a time span of 3 months between the measurements may be too short to find substantial environmental effects on the development of aggression in residential youth care. However, it should be kept in mind that the majority of adolescents placed in custody in a juvenile correctional institution spend no longer than 3 months in a residential institution and even such a short period of time in a residential institution might be experienced as a major life event for most adolescents (Van der Helm 2011).

A fourth limitation is that both repression and aggression were assessed by using adolescent self-report instruments only, which was not supplemented with independent objective observations or assessment of the perspective of prison staff and group workers. Finally, there are more aspects of adolescents’ individual functioning that may influence aggression, such as empathy, cognitive distortions, moral judgment and self-conscious emotions (Orobio de Castro et al. 2002; Spruit et al. 2016; Stams et al. 2006; Van der Helm et al. 2013; Van Langen et al. 2014), which were not addressed in this article because we did not have access to this information. Also, in further research more contextual variables should be assessed in order to be able to fully account for the effects of environmental factors.

Despite the limitations of this study, this is one of the few (short-term longitudinal) studies examining the development of aggression in adolescents in residential youth care in the Netherlands from the perspective of the importation and deprivation model. The worrisome stability of aggression asks for effective evidence-based treatment. Both Strijbosch et al. (2015) and De Swart et al. (2012) conducted a meta-analysis showing that residential youth care might reduce psychopathology, including aggressive behavior if adolescents receive evidence-based treatment, that is, ‘structured and often manualized interventions based on empirically supported theories about what causes and maintains problems, which have been proven to be effective (to some degree) in (quasi-) experimental research’ (Strijbosch et al. 2015, p. 215). However, it is plausible to suggest that evidence-based treatment during residential care is not sufficient to establish positive outcomes that generalize over context and time for juveniles with a history of adverse care and multiple risks (Lane et al. 2005). Most of them may need aftercare (James et al. 2013). Future studies should examine if, and to what extent, group climate—including the group atmosphere among adolescents, opportunities for growth, support from staff and repression—could have a moderating effect on evidence-based treatment targeting aggression of adolescents in residential institutions.
